# An unusual intra-abdominal foreign body

**DOI:** 10.11604/pamj.2014.18.176.4821

**Published:** 2014-06-24

**Authors:** George Sarin Zacharia, Thazhath Mavali Ramachandran

**Affiliations:** 1 Department of Medical Gastroenterology, Government Medical College, Calicut, Kerala, India

**Keywords:** Colon, foreign body, pig tailed stent

## Image in medicine

A 48 year old female presented to causality with high grade fever with rigors and chills, right upper abdominal pain and jaundice of one day duration. She had a past history of similar episode one month back for which she had undergone endoscopic biliary drainage. Hemogram revealed leucocytosis with polymorphonuclear cell predominance. Liver function tests; total bilirubin 8.4mg/dl, direct bilirubin 4.9mg/dl, alanine aminotransferase 96 U/L, aspartate aminotransferase 84 U/L, alkaline phosphatase 438 U/L and normal albumin 4.1gm/dl. Serum electrolytes and renal function tests were within normal limits. Plain x-ray abdomen revealed a radio-opaque foreign body in the left side of abdomen. Ultrasound abdomen revealed a mildly enlarged liver with dilated intrahepatic biliary tree, multiple gall stones and a dilated common bile duct. A provisional diagnosis of distally migrated double pigtailed biliary stent and cholangitis was made. Patient was managed with urgent endoscopic retrograde cholangio-pancreatography with stenting and started on parenteral antibiotics as well as other supportive measures. On the second day of admission stent passed off with fecal matter and repeat abdominal x-ray revealed no foreign body in the colon. Patient improved with treatment and is being planned for cholecystectomy. Stent migration is one of the complications of biliary stenting. Distal migration is more common than proximal migration. Risk of migration is more for plastic (5-10%) and covered metallic stents (3-12%) compared to uncovered metallic stents (<1%). Complications of stent migration include recurrence of cholangitis, pancreatitis and rarely intestinal obstruction or perforation. Stent modifications like creation of pig tails (single or double) or side flaps have been tried to reduce the risk of stent migration but with limited success. With the improvements in endoscopic techniques and expertise biliary stenting is more frequently done in the setting of acute cholangitis. Distally migrated stents may be identified as an intra-abdominal foreign body before it passes off in the stool. We hope this clinical image will introduce the primary care physicians to the radiological appearance in a case of distally migrated double pig tailed biliary stent.

**Figure 1:**
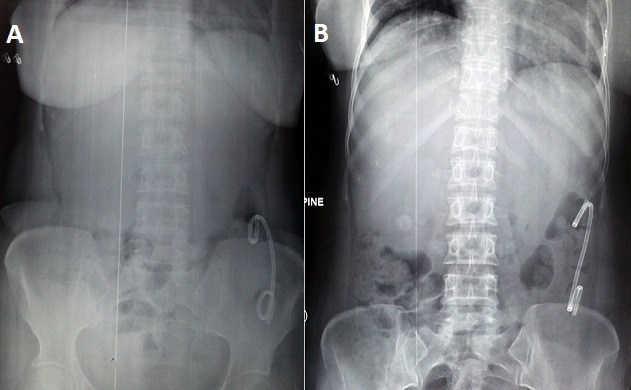
A, B) Plain x-rays of abdomen showing radio-opaque double pig tailed stent in left colon

